# Evaluation of mTOR, NFκB and BCL-2 Inhibitor Activity In Vitro in Karpas 1106P, a Primary Mediastinal B-Cell Lymphoma Cell Line

**DOI:** 10.3390/hematolrep18020025

**Published:** 2026-03-24

**Authors:** Agata Majchrzak, Sylwia Mańka, Barbara Cebula-Obrzut, Paweł Robak, Damian Mikulski, Magdalena Witkowska

**Affiliations:** 1 Department of General Hematology, Copernicus Memorial Hospital, 93-513 Lodz, Poland; majchrzak_agata@o2.pl (A.M.); sylwiamanka@umed.lodz.pl (S.M.); barbara_cebula@wp.pl (B.C.-O.); 2 Department of Hematology, Medical University of Lodz, 90-419 Lodz, Poland; pawel.robak@umed.lodz.pl; 3 Department of Hematooncology, Copernicus Memorial Hospital, 93-513 Lodz, Poland; damain.mikulski@umed.lodz.pl; 4 Department of Biostatistics and Translational Medicine, Medical University of Lodz, 90-419 Lodz, Poland

**Keywords:** mTOR inhibitors, NFkB inhibitors, BCL-2 inhibitors, primary mediastinal B-cell lymphoma

## Abstract

**Introduction**: PMBCL is an aggressive type of lymphoma characterized by high heterogeneity in clinical, molecular, and genetic features. In PMBCL, disturbances in the NFkB pathway and deregulation of BCL-2 and mTOR family proteins are observed, which may contribute to impaired apoptosis. Therefore, many strategies have been established to target the functioning of these pathways. Early clinical trials of mTOR, NFkB and Bcl-2 inhibitors suggest their activity in many hematological cancers, but their activity as monotherapy agents may still be insufficient; therefore, combinations of these compounds with other molecules acting on those active in a given cancer subtype are being sought. **Materials and Methods**: In vitro studies were conducted on a single PMBCL cell line, Karpas 1106P. We administered three novel drugs: AZD2014 (vistusertib), an inhibitor of the serine-threonine kinase mTOR; IMD-0354, an NFκB inhibitor; and ABT-199 (venetoclax), a highly selective inhibitor for BCL-2. Drugs were administered alone, in pairs and in combination of all three agents. **Results**: Based on the results of our own research, for the Karpas cell line individually, ABT-199 had the strongest pro-apoptotic effect on cancer cells, while in pairs the most potent induction of apoptosis occurred following treatment with AZD2014+ABT-199. The combination of three drugs did not have a stronger effect than either a single drug used alone or any two-drug combination. **Conclusions**: These results provide preliminary in vitro evidence that targeting the BCL-2 and mTOR pathways may enhance pro-apoptotic activity in a PMBCL cell model; however, further validation in additional cell lines and in vivo models is needed before translational implications can be considered.

## 1. Introduction

Primary mediastinal B-cell lymphoma (PMBCL) was identified as a separate disease entity within the diffuse large B-cell lymphoma (DLBCL) group of lymphomas in the WHO classification [1]. This lymphoma is located in the mediastinum and often infiltrates surrounding tissues. At diagnosis, patients are often in poor clinical condition; approximately half of them have superior vena cava syndrome and dyspnea [2]. Gene expression profile analysis confirmed differences between DLBCL and PMBCL [3]. Somatic hypermutations of the immunoglobulin and BCL-6 genes are characteristic of this lymphoma; however, the region in which the BCL-6 gene mutations occur differs from that in DLBCL and follicular lymphoma [4]. PMBCL, like DLBCL, originates from B lymphocytes. Genetic abnormalities are also present in this lymphoma, the most characteristic of which include extra copies of genes on chromosome 9p24, including Janus kinase (JAK2) and programmed death receptor ligand 1 and 2 (PD-L1, PD-L2), and chromosome 2p amplification involving cREL [5]. This abnormality occurs in almost 45% of patients with the PMBCL subtype and is much less common in patients with the activated B-cell (ABC) subtype (11%) and the germinal center B-cell (GCB) subtype (7%) [6,7].

A crucial aspect for patients with PMBCL is deciding on the treatment regimen. R-CHOP14 chemotherapy used along with consolidative radiotherapy is effective for low-risk patients but may prove insufficient for high-risk patients [8]. One chemotherapy regimen used and showing promising results is dose-adjusted etoposide, prednisone, vincristine, cyclophosphamide, and doxorubicin (DA-EPOCH-R) without radiotherapy. Relapse in PMBCL typically occurs between 12 and 18 months after treatment completion [9]. The optimal treatment approach has not yet been established. Disease recurrence may be limited to the mediastinum or involve extra-nodal sites such as the liver or kidneys. In cases of limited disease, chemotherapy with radiotherapy and auto-SCT are typically considered. However, for PMBCL, which also involves extra-mediastinal sites, high-dose chemotherapy is crucial, and allogeneic bone marrow transplantation should be considered [10].

PMBCL frequently exhibits dysregulation of the nuclear factor kappa B (NFκB), B-cell lymphoma 2 (BCL-2) and mammalian target of rapamycin (mTOR) pathways, which contribute to impaired apoptosis [11]. MTOR signaling, particularly through mTORC1 and mTORC2 complexes, is often upregulated in B-cell malignancies, driving tumor growth and metabolism. Genes overexpressed in PMBCL include those involved in the JAK-STAT pathway (IL13RA, JAK2, and STAT1), as well as key components of NFκB activation (TRAF1 and TFNAIP3), whereas genes involved in B-cell receptor signaling are downregulated. Unlike normal B-cells, PMBCL cells have a permanently “on” NF-κB pathway, which causes growth and survival of the lymphoma cells. PMBCL is often characterized by high BCL2 expressions. While PMBCL shares features with Hodgkin lymphoma (HL), BCL2 is frequently positive by immunohistochemistry, acting as an anti-apoptotic protein that promotes cell survival. Therefore, many strategies have been established to target the functioning of these pathways. The discovery of small-molecule inhibitors has become crucial to achieving desired clinical effects.

ABT-199 (venetoclax) is a highly selective inhibitor for the BCL-2 protein. It has a 3-fold-lower affinity for another protein from the BCL-2 family, BCL-XL [12]. The main function of BCL-2 is to restore the apoptosis process. ABT-199 was synthesized by Souers and colleagues. It is a selective inhibitor of the BCL-2 protein, classified as a BH3 mimetic. It binds directly to the BH3-binding groove of BCL-2, displacing pro-apoptotic proteins from this site, causing increased mitochondrial membrane permeability and subsequent activation of caspase-9, leading to apoptosis. Venetoclax binds to BCL-XL or BCL-W only at higher doses; it does not exhibit affinity for the MCL-1 protein [12,13]. Following oral administration, maximum concentrations are achieved after approximately 5–8 h. Venetoclax binds to plasma proteins and is metabolized by cytochrome P430 3A (CYP3A) enzymes [14]. Currently, venetoclax is approved for the treatment of patients with chronic lymphocytic leukemia (CLL) and acute myeloid leukemia (AML). The drug is used both as monotherapy and in combination with other drugs, including obinutuzumab (an anti-CD20 antibody) for B-cell lymphomas and azacitidine for AML.

AZD2014 (vistusertib) is an inhibitor of the serine-threonine kinase mTOR, but unlike rapamycin and its derivatives, it blocks the action of both the mTORC1 and mTORC2 complexes. It has shown broad antiproliferative effects in various cancer cell lines and tumor models, both in vitro and in vivo. AZD2014 is being studied in clinical trials, often in combination with other cancer therapies, for diseases such as recurrent meningiomas and certain types of breast and lung cancers [15]. AZD2014 was tested against a number of PI3K family enzymes and showed more than a 1000-fold selectivity against all PI3K isoforms [16].

**Figure 1:**
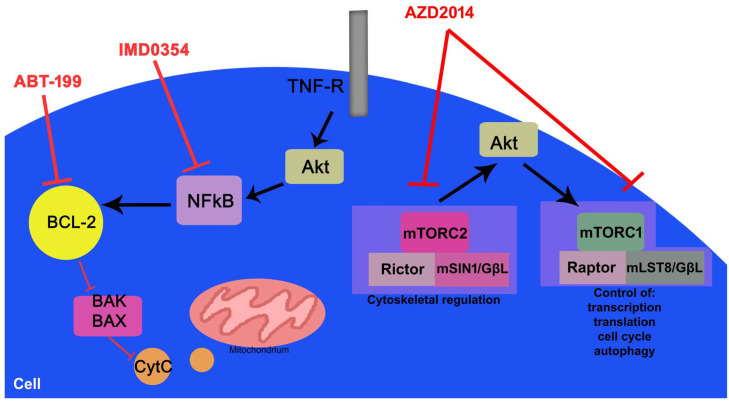
Pathways targeted by treatments currently in development for patients with DLBCL.

IMD-0354 is an NFκB inhibitor that acts by directly blocking IKKβ phosphorylation. Inhibition of NFκB activity decreases the expression of cyclin D3 and the phosphorylation of pRb, leading to cell cycle arrest and apoptosis [17]. Interruption of c-kit receptor signals abolished the DNA-binding activity of NFκB, indicating that NFκB may play a critical role in the neoplastic proliferation. IMD-0354 suppresses neoplastic proliferation of human cells with constitutively activated c-kit receptors. It has been shown to suppress cancer cell growth and promote apoptosis in culture. The drug selectivity is increased, but there are still challenges to clinical applications. Its anticancer effects are described as inhibiting cell viability and enhancing the action of chemotherapeutic agents used in treatment. It exerts anti-inflammatory effects by blocking the NFκB pathway and cytokine production [18]. It has exhibited therapeutic effects on various disease models in different organs. IMD-0354 has been proven to attenuate uveoretinitis, suppress corneal inflammation and angiogenesis, and restore the barrier function of different human cells. The mechanisms of action of these three inhibitors is shown in Figure 1.

Karpas 1106-P is a well-characterized PMBCL cell line and was selected as a relevant model to evaluate the cytotoxic effect of mTOR (AZD2014), NFκB (IMD-0354), and BCL-2 (ABT-199) inhibitors in monotherapy and in combinations of two and three inhibitors. To explore potential interactions between survival and anti-apoptotic pathways, we investigated the alone and combined effects of vistusertib, IMD-0354, and venetoclax on apoptotic activity, while overall caspase pathway engagement was monitored as a general indicator.

## 2. Materials and Methods

*Cell line.* Human PMBCL, i.e., the Karpas 1106P cell line (DSMZ; Leibniz, Germany), was cultured in RPMI1640 medium supplemented with 20% heat-inactivated fetal bovine serum, 2 mM L-glutamine and antibiotics (penicillin 50 IU/mL/streptomycin 50 µg/mL) (all PAN-Biotech, Aidenbach, Germany) under standard conditions: 37 °C, 5% CO_2_, fully humidified.

*Reagents.* Stock solutions (10 mM) of AZD2014, IMD-0354 and ABT-199 (all from Selleckchem, Houston, TX, USA) were prepared by dissolving each reagent in DMSO (Sigma-Aldrich, St. Louis, MO, USA) and stored at −20 °C. To obtain the required concentrations of reagents, dilutions in RPMI1640 were made. Control cells were treated with DMSO at a concentration not exceeding 0.1%, corresponding to the solvent in drug-treated samples.

*Cytotoxicity assay.* Cell viability was determined by propidium iodide (PI) staining. Briefly, cultures were washed twice in cold 1% PBS (PAN-Biotech, Aidenbach, Germany) and stained with 10 µg/mL PI (Sigma Aldrich, Burlington, MA, USA) for 15 min, at room temperature, in the dark. Then, the cell fluorescence was measured using flow cytometry with a red FL-3 fluorescent filter and the percentage of PI-positive cells was defined as non-viable. In a preliminary experiment, cells were incubated with tested inhibitors at concentrations reaching 200 µM (AZD2014, IMD-0354) or 100 µM (ABT-199) for 24 and 48 h. The above time points were chosen to evaluate early and intermediate apoptotic effects. Based on these results, the lowest concentrations of reagents that induced significant cytotoxic effect compared with controls were selected for further analyses. In the final experiments, cells (0.5 × 10^6^/mL) were incubated under sterile conditions in 75 mL dishes (Nunc, Roskilde, Denmark) with the following concentrations of tested inhibitors: AZD2014 (5 µM), IMD-0354 (10 µM) and ABT-199 (50 nM). Furthermore, the combinations of inhibitors in the culture sets were prepared as follows: AZD2014+IMD0354, AZD2014+ABT-199, ABT-199+IMD0354, and the triple combination of AZD2014+IMD0354+BCT-199.

To identify the optimal dose for subsequent experiments, cytotoxicity data obtained for multiple concentrations of a single drug were analyzed using Dunnett’s test, with comparisons made against the control.

*Apoptosis assay.* Apoptosis was evaluated by measuring phosphatidylserine externalization using an Annexin V Apoptosis Detection Kit (Becton Dickinson, San Jose, CA, USA). Following incubation, cells were washed twice with cold PBS and re-suspended in 85 μL of binding buffer. Annexin V–FITC (5 μL) and PI (10 μL, 10 μg/mL) were added, followed by incubation for 15 min in the dark, and then the fluorescence was measured by flow cytometry using an FL-1 filter. The total level of apoptosis was determined by summing the percentage of early (Annexin-V+/PI-) and late apoptotic (Annexin-V+/PI+) cells in each sample.

*Assessment of mitochondrial membrane potential (ΔΨ).* The loss of the mitochondrial membrane potential was detected using MitoTracker™ Red CMXRos (MolecularProbes, Eugene, OR, USA). The 1 mM stock solution of the reagent was diluted in the culture medium to a final working concentration of 50 nM, and 2.5 mL was added to the cell cultures. Following a 15 min incubation (humidified atmosphere with 5% CO_2_ at 37 °C), the drop in the ΔΨ was measured by flow cytometry with a fluorescent filter—FL3.

*Expression of caspase-3, -8, and -9.* After incubation, the cells were fixed and permeabilized using a Cytofix/CytopermTM solution for 20 min on ice. Subsequently, cells were washed twice and re-suspended in Perm/WashTM buffer with anti-caspase-3 antibodies (all from BD Pharmingen, San Diego, CA, USA). After a 30 min incubation at room temperature, the cells were washed in Perm/WashTM buffer and immediately analyzed using flow cytometry with a fluorescent filter—FL1. Caspase activation was assessed using complementary approaches, including cleavage-based detection of caspase-3 and enzymatic activity assays for caspases-8 and -9. Therefore, all our conclusions will be limited to overall caspase involvement in apoptosis rather than to the relative contribution of individual caspases. In order to detect caspase-8 and -9 activity, FAM-LETD-FMK FLICA^®^ Caspase 8 and FAM-LEHD-FMK FLICA^®^ Caspase 9 Assay Kits were used (Immunochemistry Technologies, Davis, CA, USA). All assays were performed according to the manufacturer’s instructions, and the results were evaluated by flow cytometry.

*Flow cytometry analysis.* All fluorescence measurements were performed using flow cytometry (FACSCanto II, Becton Dickinson) using standard emission filters: green for FITC (FL1, λ = 530 ± 20 nm), orange for PE (FL2, λ = 560–600 nm), and red for Cy-5 (FL3, λ > 600 nm), where necessary. Ten thousand cells per sample were acquired for each analysis.

*Statistical analysis.* All analyses were performed using GraphPad Prism 9.5 (San Diego, CA, USA). The normality of the data distribution was checked using the Shapiro–Wilk test. Significant differences between the treatment groups were analyzed using a one-way ANOVA test followed by a post hoc Tukey test, and *p* < 0.05 was considered as statistically significant. In the figures, the data are presented as the mean ± standard error of the mean (SEM) of five independent experiments and significance between the studied groups is marked as follows: ns > 0.05; * *p* < 0.05; ** *p* ≤ 0.01; *** *p* ≤ 0.001; **** *p* ≤ 0.0001.

## 3. Results

**Figure 2:**
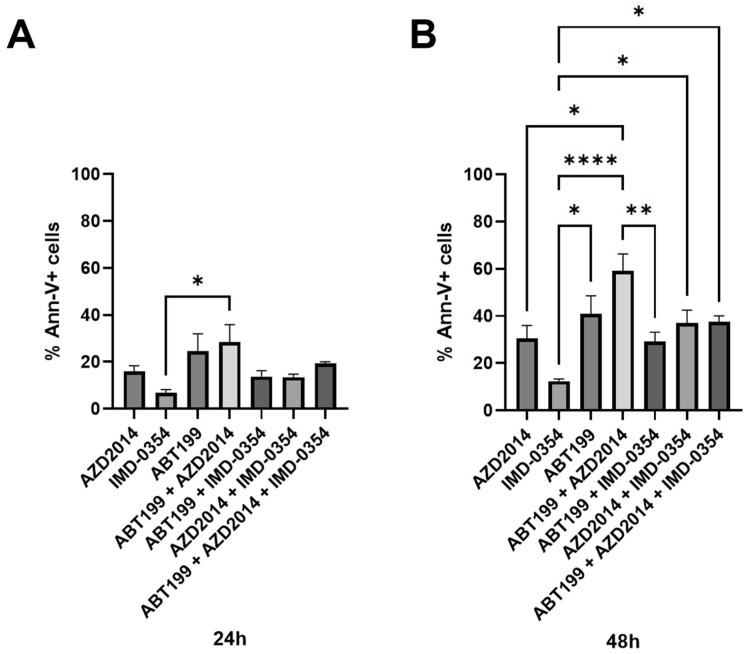
Pro-apoptotic effect of AZD2014 (5 µM), IMD-0354 (10 µM) and ABT-199 (50 nM) used alone and in double combinations of AZD2014+IMD-0354, AZD2014+ABT-199 and AZD2014+ABT-199 or in triple combination of AZD2014+IMD-0354+ABT-199 was determined by an Ann-V/PI assay as described in Materials and Methods. Percentage of total apoptotic cells after 24 h (**A**) and 48 h (**B**) of culture in the Karpas cell line.

In 24 h cultures, no significant differences in apoptosis induction were observed among the tested inhibitors, whether administered individually, in dual combinations, or as a triple-drug regimen (Figure 2A). In contrast, in 48 h cultures, among the individually used inhibitors, the strongest ability to induce apoptosis was observed for ABT-199 with an average of 40.9% ANX-V-positive cells. In pairs, AZD2014+ABT-199 had the highest level of apoptosis (59.1%), which was significantly greater than the effects achieved with either AZD2014 used alone (12.3%) or ABT-199+IMD-0354 (29.2%); however, the nature of this interaction was not formally assessed. In turn, the combination of AZD2014+IMD-0354+ABT-199 did not have a stronger pro-apoptotic effect compared with either drug used alone or AZD2014+ABT-199 (Figure 2B).

**Figure 3:**
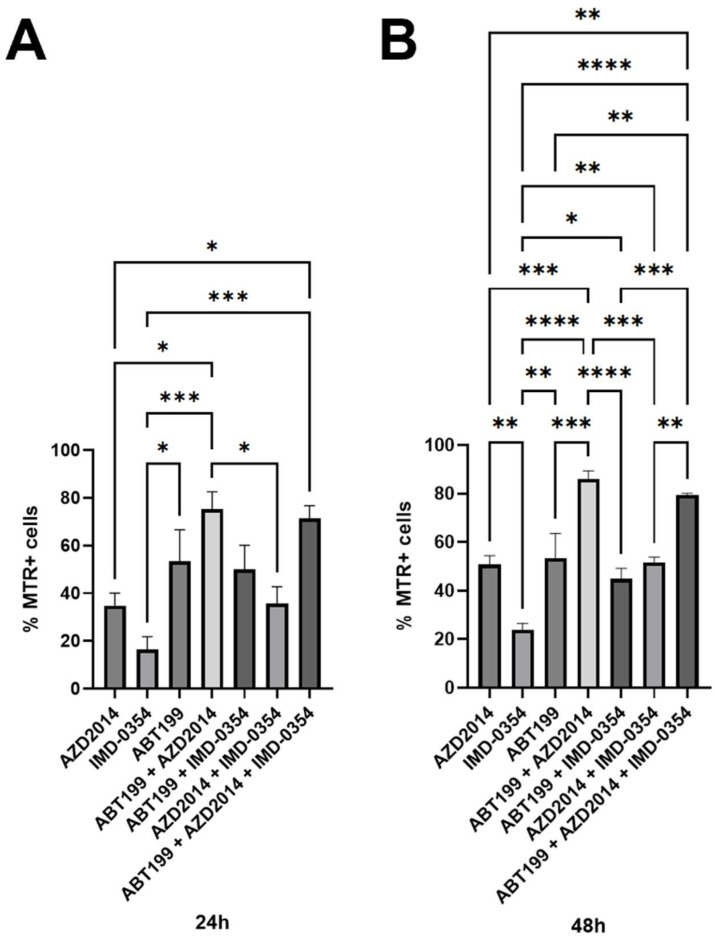
Mechanisms of pro-apoptotic activity of AZD2014 (5 µM), IMD-0354 (10 µM) and ABT-199 (50 nM) used alone and in double combinations of AZD2014+IMD-0354, AZD2014+ABT-199 and AZD2014+ABT-199 or in triple combination of AZD2014+IMD-0354+ABT-199. Each bar represents the mean ± SEM percentages of cells with a decline in the mitochondrial membrane potential (ΔΨ) after 24 h (**A**) and 48 h (**B**) of culture in the Karpas cell line.

**Figure 4:**
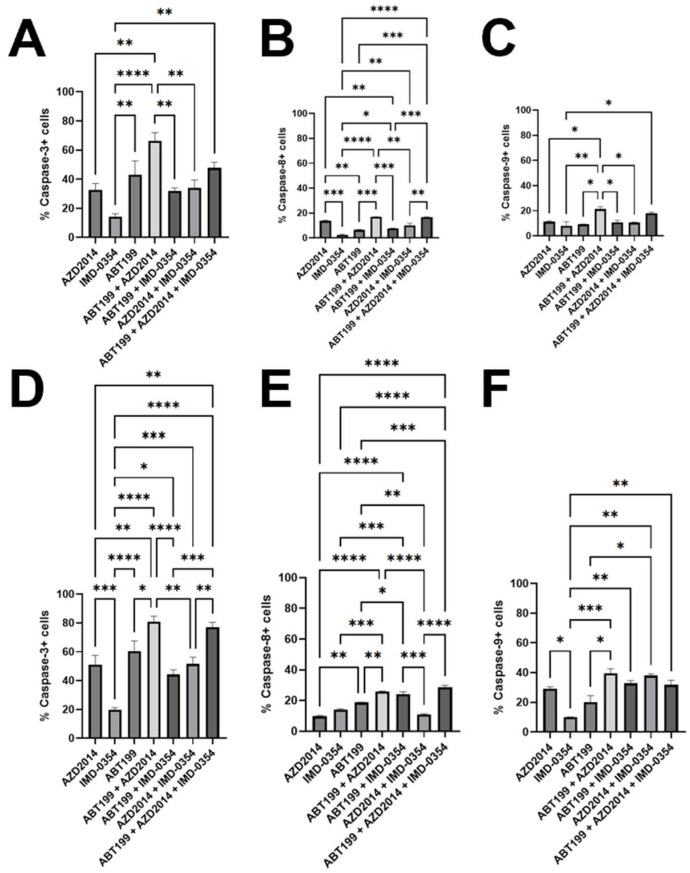
Mechanisms of pro-apoptotic activity of AZD2014 (5 µM), IMD-0354 (10 µM) and ABT-199 (50 nM) used alone and in double combinations of AZD2014+IMD-0354, AZD2014+ABT-199 and AZD2014+ABT-199 or in triple combination of AZD2014+IMD-0354+ABT-199. Each bar represents the mean ± SEM percentages of positive cells with the activation of caspase-3, -8, and -9 in the Karpas cells after 24 h (**A**–**C**) and 48 h (**D**–**F**) of culture.

In both 24 and 48 h cultures, among the tested inhibitors administered as monotherapy, ABT-199 increased the percentage of cells with a decline in the ΔΨ (approximately 53.0% for both time periods) compared with IMD-0354 (24 h: 16.5%, 48 h: 23.8%). In turn, AZD2014+ABT-199 resulted in a drop in ΔΨ (24 h: 75.3%, 48 h: 86.0%); in the 48 h culture the effect was significantly higher than observed for ABT-199+IMD-0354 (44.9%) and AZD2014+IMD-0354 (51.7%). The triple combination of inhibitors showed no significant differences compared with the combination of ABT-199+AZD2014 (Figure 3A,B). The above-presented results of the effect of tested inhibitors on the decrease in ΔΨ are consistent with the results of the apoptosis assay. ABT-199 elicited the most robust caspase-3 activation in the 24 and 48 h cultures, but with differences compared only with IMD-0354 (Figure 4A,D). No significant differences were observed between the effects of the tested inhibitors used alone on the activation of caspases-8 and -9. The strongest induction of caspase-3 activation was observed for the combination of AZD2014+ABT-199 both in the 24 and 48 h cultures. For caspase-8, the effect was also the strongest for this combination, although it was comparable to the triple-drug combination. Caspase-9 was likewise most strongly activated by the two-drug combination of AZD2014+ABT-199 (Figure 4A–F).

Based on dose–response analyses in the Karpas cell line, concentrations inducing a statistically significant but moderate cytotoxic effect were selected to enable further mechanistic studies. For AZD2014, a significant increase in the percentage of JP^+^ cells was observed already at low concentrations (1–5 µM), whereas higher doses did not result in a proportional enhancement of the effect; therefore, 5 µM was chosen for subsequent experiments as the lowest concentration consistently inducing cytotoxicity (Supplementary Figure S1). In the case of ABT-199, the first clear and statistically significant increase in JP^+^ cells was detected at 50 nM, justifying the selection of this concentration for further analyses (Supplementary Figure S2). For IMD-0354, a pronounced dose–response relationship was observed, and the lowest concentration showing a statistically significant increase in JP^+^ cells compared with the control (10 µM) was selected for subsequent experiments (Supplementary Figure S3). Importantly, combination experiments were performed using these preselected single concentrations (fixed-dose design). This approach was intended to evaluate biological effects under mechanistically informative conditions rather than to perform formal drug-interaction analyses.

## 4. Discussion

PMBCL is a relatively rare malignancy belonging to the aggressive DLBCL group. The latest studies have elucidated the biologic hallmarks of PMBCL that are reminiscent of classical HL, including the importance of the JAK-STAT and NFκB signaling pathways, as well as an immune evasion phenotype, through multiple converging genetic aberrations [11,19]. The prognosis has improved after rituximab addition; however, whether there is a single standard therapy for all PMBCL patients and whether to combine with radiotherapy still remains controversial [8]. Regardless of the first-line treatment, refractory disease can occur in up to 10% of patients and correlates with poor outcome. The most important goal remains searching for potential novel therapeutic targets and new treatment strategies that may complement conventional therapy [20].

In recent years, increasing emphasis has been placed on modern molecularly targeted therapy in the treatment of patients with B-cell lymphomas, including the combination of drugs affecting various signaling pathways. In this work, we investigated the effects of three modern drugs—inhibitors of pathways involved in the development of PMBCL. The drugs were used as monotherapy, in dual-drug regimens, and in combination with all three drugs simultaneously on the Karpas cell line. A similar study examining the antitumor activity of such new drug combinations in vitro has not yet been published. Importantly, our experiments were performed in a single line, so the findings represent preliminary in vitro evidence and may not capture the full biological heterogeneity of PMBCL.

The mechanism of action of ABT-199 (venetoclax) is primarily based on the inhibition of the anti-apoptotic activity of BCL-2 family proteins. It is a BH3-only mimetic and, due to its specific mechanism of action—the “so-called” depression model—it incorporates itself into the BCL-2-interacting mediator of cell death (BIM) protein. This triggers a cascade of changes and leads to the induction of spontaneous cancer cell death. Venetoclax’s mechanism of action involves inhibiting the expression of BCL-2 proteins and inducing apoptosis of lymphoma cells, preventing their accumulation in lymph nodes, extra-lymphatic organs, bone marrow, and spleen. In the Karpas cell line, venetoclax demonstrated the strongest antitumor activity, both alone and in combination with AZD2014. The combination of the three drugs did not influence the enhanced pro-apoptotic effect. This indicates that venetoclax is by far the most effective in vitro of the drugs tested in this work. In our study, no direct assessment of on-target pathway inhibition was performed, i.e., phosphorylation status of mTORC1/2 downstream targets, NFκB nuclear activity, or BCL-2 target engagement markers; therefore, mechanistic interpretations regarding combined mTOR and BCL-2 inhibition are based on functional apoptotic readouts and should be considered as hypothesis-generating. The induction of apoptosis by venetoclax was confirmed in 2013 by Soures et al. [12]. In their study, they described significant features indicative of apoptosis initiation. A single dose of ABT-199 administered to three patients with refractory CLL resulted in tumor cell lysis within 24 h. These data indicate that selective pharmacological inhibition of BCL-2 may be an effective treatment option in patients diagnosed with BCL-2-dependent lymphoproliferative disorders.

After initial success in patients with CLL, venetoclax is currently being intensively studied in patients with NHL, including PMBCL, in both first and subsequent lines of treatment. In a phase 1 study by Rutherford et al., venetoclax was administered with standard front-line therapy dose-adjusted EPOCH-R (rituximab, etoposide, vincristine, cyclophosphamide and doxorubicin) [21]. In 30 PMBCL patients, the maximum tolerated dose of venetoclax was 800 mg for 10 days and the established recommended phase 2 dose was 600 mg for 5 days due to tolerability for treatment duration. The most common grade 3–4 side effects were cytopenias (93%) and febrile neutropenia (63%). There was one treatment-related death (sepsis). Overall Response Rate (ORR) was 96.7% (95%); 28 (93%) of 30 patients had complete response (CR) and one (3%) had partial remission (PR). Addition of venetoclax to standard first-line treatment showed an acceptable safety profile and it is worth further study. According to the results, the combination is being investigated in Alliance 051701 [22] (NCT03984448).

However, despite these advances in research, venetoclax has been found to lead to varying degrees of drug resistance in the treatment of PMBCL, which also limits its potential use [23]. Resistance may involve upregulation of alternative anti-apoptotic proteins such as MCL-1 and BCL-XL, as well as activation of parallel survival signaling pathways. Clinical trials in patients diagnosed with acute myeloid leukemia have shown that the primary approach to resolving venetoclax resistance is combining it with other modern drugs, such as hypomethylating agents [24]. We observed similar results in our study, where the antitumor activity of venetoclax was most pronounced when combined with an mTOR inhibitor (AZD2014). It should be noted that our experiments were performed using a fixed-dose design, so the observed effects cannot be formally classified as synergistic. Consequently, the enhanced pro-apoptotic effects observed for selected combinations can be interpreted as increased activity under the tested experimental conditions rather than proven synergism. AZD2014 is a small-molecule, ATP-competitive mTOR inhibitor that inhibits both the mTORC1 and mTORC2 complexes and has a greater inhibitory effect on mTORC1 than clinically approved drugs. AZD2014 has broad antiproliferative effects demonstrated in multiple cell lines. In this study, a cytotoxic effect was observed when combined with venetoclax, and this effect was statistically significantly stronger compared to venetoclax used as monotherapy. Previously published studies have reported that the addition of AZD2014 in PI3Kβ/δ inhibitor-resistant models (AZD8186) overcame resistance to PI3Kβ/δ inhibition and completely prevented DLBCL cell growth in vivo and in cell-line-derived and patient-derived xenograft mouse models [25]. Ezella et al. [26] reported similar observations of enhanced cytotoxic effects in combination treatments. Based on in vitro studies, the combination of ibrutinib, a Bruton’s tyrosine kinase (BTK) inhibitor, and AZD2014 was highly synergistic in killing DLBCL cell lines of the ABC subtype. Simultaneous inhibition of BTK and mTOR induced apoptosis both in vitro and in vivo and resulted in tumor regression in a xenograft model.

However, these results were not supported by a phase 1b study by Collins et al. where vistusertib was combined with acalabrutinib (a covalent Bruton’s tyrosine kinase inhibitor) in patients with relapsed/refractory B-cell NHL [27]. Different doses of the combination administered as intermittent or continuous schedules of vistusertib were evaluated and the ORR was 12% (3 out of 25 patients). These data suggest that vistusertib does not modulate targets sufficiently to add to the clinical activity of acalabrutinib monotherapy.

On the other hand, vistusertib was investigated alone in a phase II study in relapsed, refractory DLBCL [28]. Thirty patients received vistusertib and six received vistusertib-rituximab for up to six cycles. For monotherapy, two PRs were achieved, and 19% had stable disease (SD) within six cycles. The median progression-free survival (PFS) was 1.69 months, and median overall survival (OS) was 6.58 months. Moreover, Tang et al. developed a rituximab (anti-CD20)-modified mTOR inhibitor, AZD2014, loaded into nanoparticles (Ab-NPs-AZD-2014) [29]. In a cultured NHL cell line, Ab-NPs-AZD-2014 inhibited lymphoma cell growth, induced cell apoptosis, and blocked activation of mTORC1 and mTORC2 in Raji cells. These results show that antibody modification and nanomaterial loading of AZD2014 with anti-CD20 significantly improved efficacy of AZD2014. This approach might be a promising therapeutic option for B-cell NHL patients.

The NFκB inhibitor IMD-0354 proved to be the least active, both alone and in combination with other tested drugs. IMD-0354 is a small-molecule inhibitor of IKKβ that can effectively inhibit the NFκB pathway. Furthermore, IMD-0354 can inhibit various cancer cells in culture, but its poor solubility limits its clinical application. In humans, the NFκB pathway is implicated in several biological processes, such as the development of inflammation, cell proliferation, and resistance to infection. Previously published studies have shown that IMD-0354 effectively inhibited cancer cell growth in vitro, similar to preclinical models [30]. In addition to its anti-inflammatory effects, IMD-0354 has also been reported to have cytostatic effects on cancer cells, such as breast cancer and melanoma [31]. Numerous studies have shown that NFκB activation is a hallmark of most types of human cancer, including leukemia and lymphoma [32]. For this reason, studies of IMD-0354 have also included patients with lymphatic malignancies. Kanduri et al. evaluated the effect of IMD-0354 in patients with CLL [33]. The results clearly show that IMD-0354 induced apoptosis (mean 26%, range 8–48%) in CLL cells, regardless of immunoglobulin heavy-chain variable gene (IGHV) mutation status, and demonstrated a dose-dependent cytotoxic effect. Treatment with IMD-0354 also significantly reduced NFκB DNA-binding activity in CLL cells. Furthermore, the authors identified differences in the expression levels of pro- and anti-apoptotic genes following IMD-0354 treatment. IMD-0354 has not yet been evaluated in patients with DLBCL as monotherapy or in combination with other novel agents.

Moreover, it is extremely important to consider the potential toxicity of the combination of investigated novel drugs. Toxicity in novel drug combinations is a significant challenge in pharmaceutical development, as combining agents to enhance therapeutic efficacy may result in increased or unexpected side effects. We should be aware that as investigated combinations may benefit antitumor activity, they might also lead to additive toxicity. As a result, adverse events may result, or the combination may create new or more severe adverse effects. In the future, investigating the therapeutic window will also be extremely important. This window is the range of plasma concentration between the minimum effective concentration and the minimum toxic concentration. There are drugs for which this interval is very narrow. Therefore, individualized dosing using therapeutic drug monitoring may help optimize treatment in a safe and effective manner. Importantly, these considerations are extrapolations based on existing literature and not directly evaluated in the present in vitro study. The next step should be the inclusion of additional PMBCL and DLBCL cell lines, the use of primary patient samples, and ultimately clinical trials.

To conclude, the data presented in this study provide preliminary evidence supporting rational dual therapy including the BCL-2 inhibitor and mTOR inhibitor as the most active combination in the PMBCL cell line. Unfortunately, our study is not without limitations. One of them is the use of a single Karpas cell line and the absence of direct target engagement analyses for mTOR and BCL-2 inhibition. Thus, the results should be regarded as preliminary evidence based on functional flow cytometry assays and hypothesis-generating. Further validation in additional cell lines or patient samples and with pathway-specific markers are needed.

## Data Availability

The original contributions presented in this study are included in the article/Supplementary Materials. Further inquiries can be directed to the corresponding author.

## Supplementary Materials

The following supporting information can be downloaded at: https://www.mdpi.com/article/10.3390/hematolrep18020025/s1, Supplementary Figure S1: AZD2014–induced cytotoxicity in vitro; Supplementary Figure S2: ABT-199–induced cytotoxicity in vitro; Supplementary Figure S3: IMD-0354–induced cytotoxicity in vitro.
