# Supplementary material for: Evaluation of mTOR, NFκB and BCL-2 Inhibitor Activity In Vitro in Karpas 1106P, a Primary Mediastinal B-Cell Lymphoma Cell Line
Source: Hematol Rep. 2026 Mar 24;18(2):25. doi: 10.3390/hematolrep18020025 (PMC13116572; doi:10.3390/hematolrep18020025)
Supplement: Supplementary file 1 [file hematolrep-18-00025-s001.zip › hematolrep-4115675-supplementary.pdf]

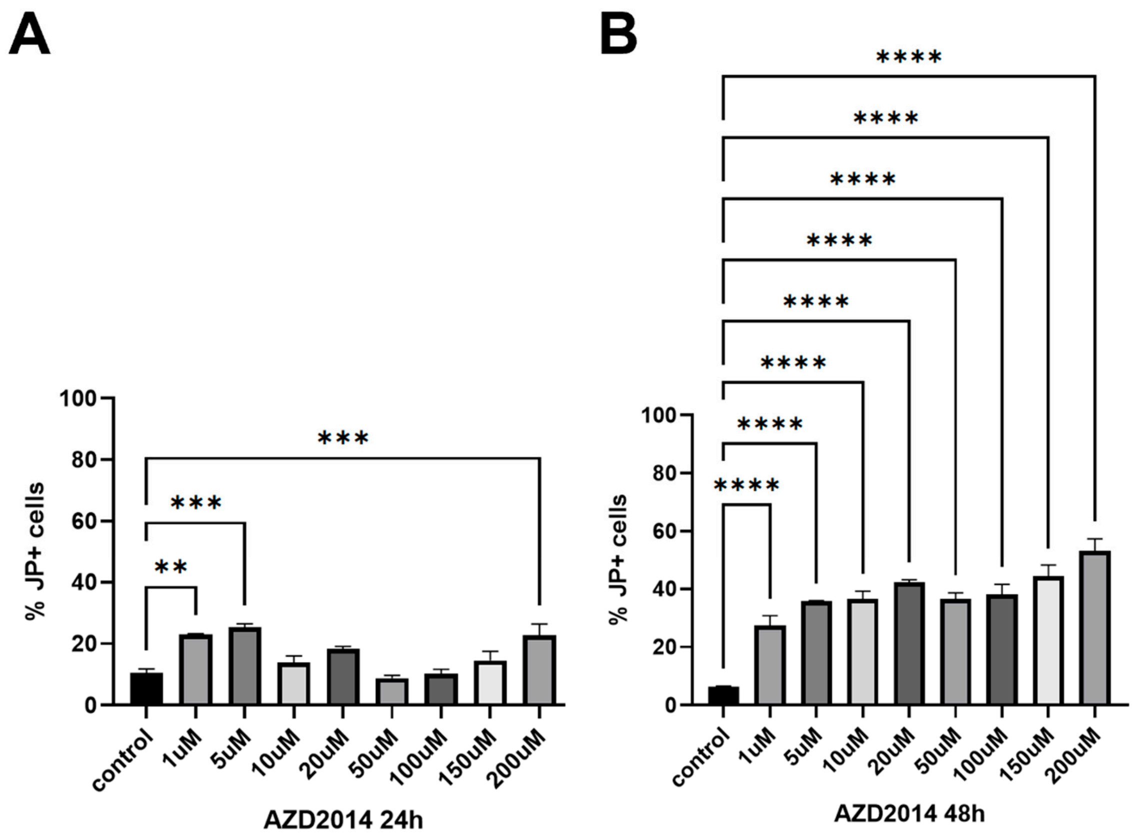

Supplementary Figure S1: AZD2014–induced cytotoxicity in vitro. Percentage of JP$^+$ cells after **(A)** 24 hand **(B)** 48 h treatment with increasing concentrations of AZD2014. Data are presented as mean ± SEM.

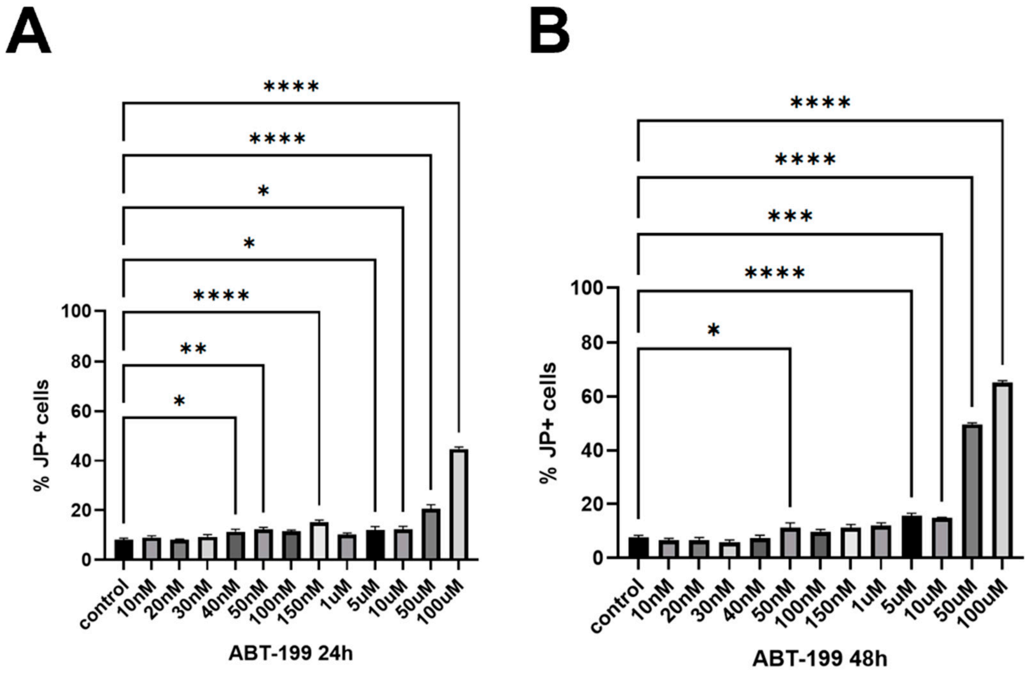

Supplementary Figure S2: ABT-199–induced cytotoxicity in vitro. Percentage of JP^{+} cells after **(A)** 24 hand **(B)** 48 h treatment with increasing concentrations of AZD2014. Data are presented as mean ± SEM.

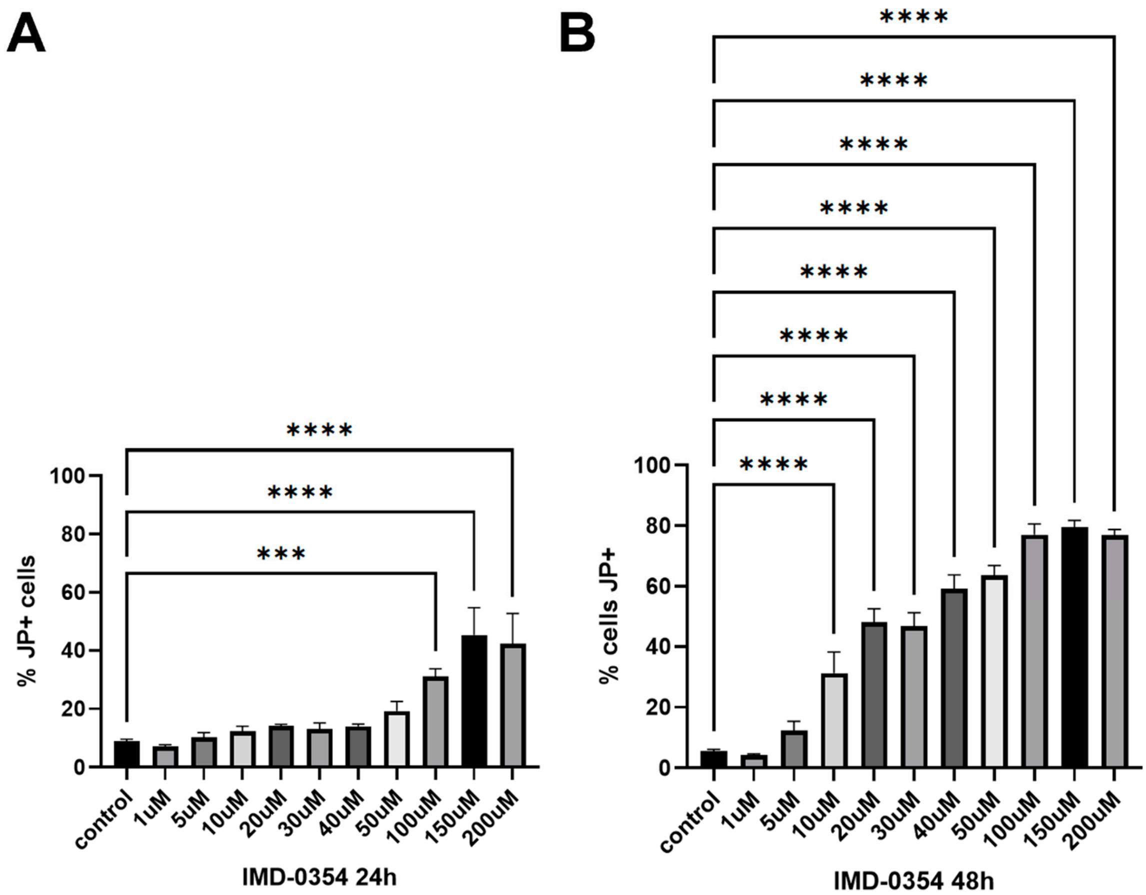

Supplementary Figure S3: IMD-0354–induced cytotoxicity in vitro. Percentage of JP^{+} cells after **(A)** 24 hand **(B)** 48 h treatment with increasing concentrations of AZD2014. Data are presented as mean ± SEM.
